# Immune and cellular determinants of response to HMA‐venetoclax regimens in acute myeloid leukemia

**DOI:** 10.1002/hem3.70498

**Published:** 2026-10-13

**Authors:** Alfredo Marchetti, Francesca Lazzaroni, Matteo Claudio Da Via', Marta Lionetti, Giulia Maggioni, Alessia Campagna, Denise Ventura, Giorgio A. Croci, Alessio Marella, Akihiro Maeda, Antonio Matera, Emanuele Calvi, Margherita Scopetti, Filippo Viviani, Nicolò Rampi, Davide Bonometti, Elena Benzoni, Manuela Zappa, Sonia Fabris, Marzia Barbieri, Stefania Pioggia, Claudia Leoni, Antony Ricchiuti, Matteo G. D. Porta, Mariarita Sciumè, Francesco Passamonti, Nicola S. Fracchiolla, Niccolò Bolli

**Affiliations:** ^1^ Department of Oncology and Hematology‐Oncology University of Milan Milan Italy; ^2^ Hematology Unit, Fondazione IRCCS Ca' Granda Ospedale Maggiore Policlinico Milan Italy; ^3^ IRCCS Humanitas Research Hospital Rozzano Milan Italy; ^4^ Department of Pathophysiology and Transplantation University of Milan Milan Italy; ^5^ Division of Pathology, Fondazione IRCCS Ca' Granda Ospedale Maggiore Policlinico Milan Italy; ^6^ Clinical Pathology Unit, Fondazione IRCCS Ca' Granda Ospedale Maggiore Policlinico Milan Italy; ^7^ Department of Biomedical Sciences Humanitas University, Pieve Emanuele Milan Italy

## Abstract

Hypomethylating agents (HMAs) combined with Venetoclax have reshaped the therapeutic landscape of acute myeloid leukemia (AML), yet the biological determinants of response remain unclear. We integrated clinical and genomic profiling in 132 newly diagnosed or relapsed/refractory AML patients treated with HMA‐Venetoclax and performed single‐cell RNA sequencing and T cell receptor (TCR) profiling in 10 newly diagnosed cases to define cellular and immune correlates of response. Response rates and survival aligned with published data. DNA Sequencing confirmed the favorable impact of IDH2 and the poor prognosis of TP53 mutations. scRNA‐seq and TCR analysis showed that responders exhibited expansion of cytotoxic CD8^+^ T cells, increased TCR clonal diversity, and activation of antigen‐presentation pathways. Nonresponders displayed baseline inflammatory signaling, impaired myeloid maturation, and a suppressive microenvironment enriched in IDO1^+^ dendritic cells. Resistant AMLs showed stem‐like dedifferentiation and sustained EMT. LAG‐3 expression marked dysfunctional T cell states and emerged as a potential biomarker and therapeutic target.

## Graphical abstract

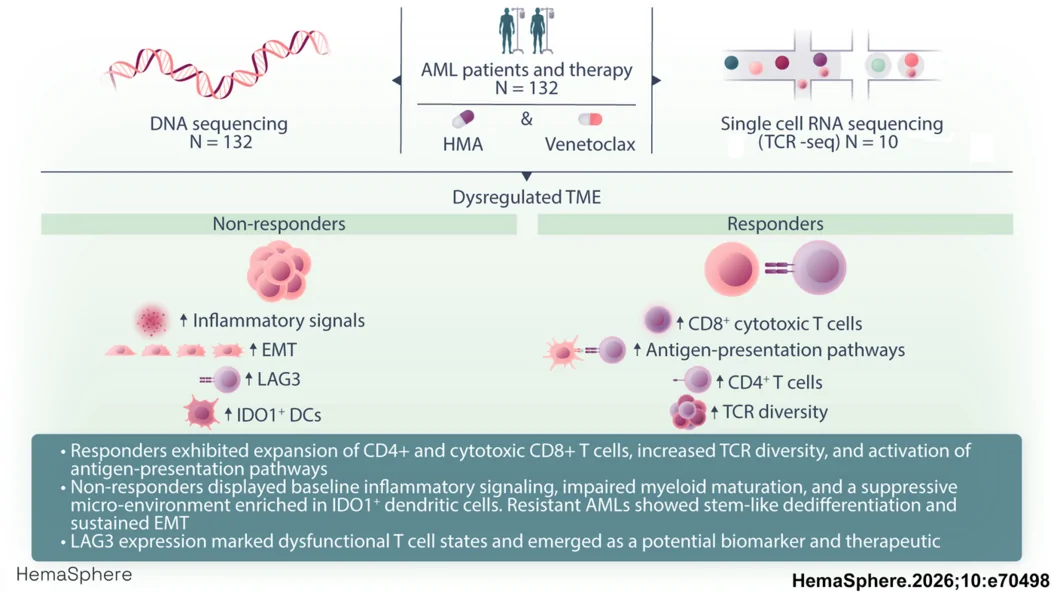

## INTRODUCTION

Acute myeloid leukemia (AML) is a genomically heterogeneous malignancy stemming from maturation arrest and proliferative advantage of neoplastic myeloid‐primed clones. While intensive chemotherapy and, more recently, lower‐intensity regimens such as hypomethylating agents (HMAs) combined with the BCL2 inhibitor venetoclax (HMA‐Ven) have significantly improved response rates, including in older or unfit patients, long‐term remissions remain uncommon, and resistance is frequent.^1^

The mechanisms driving relapse and therapeutic failure in AML are multifactorial. In addition to intrinsic leukemic alterations, tumor microenvironment (TME) and immune dysfunction have emerged as key contributors. In this scenario, chronic interferon‐γ (IFN‐γ) signaling, particularly in TP53‐mutated AML, has been associated with immune exhaustion, resistance to genotoxic stress, and adverse outcomes. Single‐cell and bulk RNA sequencing (scRNA‐seq) studies have highlighted enrichment of dysfunctional T‐cell subsets, including exhausted and regulatory phenotypes, especially in genomically high‐risk disease, particularly in the setting of patients treated with intensive chemotherapy.^2^, ^3^, ^4^, ^5^, ^6^, ^7^, ^8^

In older and/or unfit patients, HMAs combined with the BCL2 inhibitor venetoclax have transformed frontline therapy, achieving high remission rates even in adverse‐risk subsets. While deep responses can be achieved through nonintensive regimens, remissions are often short‐lived, and relapse is often observed.^9^, ^10^, ^11^

Resistance to HMA‐Ven therapy involves diverse molecular mechanisms, such as upregulation of alternative anti‐apoptotic proteins (i.e., MCL1), BAX mutations, and mutations in TP53, NRAS, and FLT3.^12^, ^13^, ^14^ However, the contribution of immune dysregulation to venetoclax resistance remains poorly defined.

To this end, we performed a combined bulk genomic and single‐cell transcriptomic analysis in a cohort of AML patients treated with HMA‐Venetoclax, focusing on early treatment timepoints to dissect the molecular and immunological determinants of response.

## MATERIALS AND METHODS

### Patients and treatment regimen

We retrospectively analyzed 132 patients with AML treated with HMAs plus venetoclax. For single‐cell transcriptomic profiling, a subset of 10 newly diagnosed de novo AML patients treated at Fondazione IRCCS Ca' Granda Ospedale Maggiore Policlinico was selected. The study was approved by the Institutional Review Board (*protocol IRB 439_2022bis*) and conducted in accordance with the Declaration of Helsinki. Written informed consent was obtained, and samples were anonymized prior to analysis. Bone marrow (BM) aspirates were collected at diagnosis (TP1) and post‐cycle one (TP2; median Day 27, range 24–51). For the retrospective cohort, response was assessed according to the European Leukemia Network criteria for response. Patients who underwent single‐cell RNA‐Sequencing were instead grouped into three categories: Responders (CR/CRi), Early relapse (ER) if they initially responded at TP2 but then failed treatment within the first 4 cycles of treatment, or Refractory (RR) if disease was still present and active at sampling time TP2. ER and RR patients were collectively defined as “non‐responders” (NR) in binary contrasts.

### Sample processing and genomic profiling

BM aspirates were processed via Ficoll‐Paque density gradient centrifugation, followed by red blood cell lysis and PBS washes. Mononuclear cells (MNCs) were cryopreserved in 90% fetal calf serum and 10% dimethyl sulfoxide. Genomic DNA was extracted from MNCs and sequenced using a custom 73‐gene panel targeting recurrent mutations in myeloid malignancies. Libraries were prepared using the KAPA HyperPlus kit (Roche) and sequenced on Illumina MiSeq (150/200 bp reads, paired‐end configuration). Variant calling employed Mutect2, LoFreq, VarDict, FreeBayes, and Strelka; variants detected by ≥2 callers were retained. The presence of NPM1, IDH1, IDH2, FLT3‐ITD, NRAS, and KRAS mutations was taken into account when assigning patients to ELN2024 classes. Information on DDX41 mutations was not available for the whole cohort, and among cases that were tested, no mutated cases were observed.

### Single‐cell RNA and TCR sequencing

Thawed MNCs were processed using the Chromium Next GEM Single Cell 5′ v2 kit (10× Genomics), loading ~9000 cells/sample. Libraries for gene expression and TCR enrichment were generated and sequenced on the Illumina NovaSeq 6000 platform (150 bp paired‐end reads), targeting 100,000 reads/cell for gene expression and 15,000 reads/cell for TCR.

### Preprocessing and data analysis

Raw data were processed with Cell Ranger v5.0 and aligned to GRCh38. Ambient RNA was removed using SoupX, and cells were filtered based on mitochondrial content (<10%), gene count (200–3000) (Supporting Information S1: Figure 1), and doublet exclusion (DoubletFinder v2.0.4) (preprocessing quality assessments can be found in Supporting Information S1: Figure 1). Cell types were annotated using the Bone Marrow Reference Map v4.4.0 and ProjecTILs atlas. Data integration and dimensionality reduction were performed using Seurat v4.4.0 and Harmony v1.2.0. Differential expression was assessed via the Wilcoxon rank‐sum test; GSEA was conducted using clusterProfiler and MSigDB Hallmark sets. EMT module scores were calculated using GSVA, with a high‐EMT cutoff set at 0.39. TCR clonotype and diversity metrics were analyzed using scRepertoire v2.0.^15^ CNV inference on single‐cell RNA‐seq data was obtained using Numbat v1.5.1 following the standard workflow available at https://github.com/kharchenkolab/numbat.

### Data visualization

Data visualization employed ggplot2, ComplexHeatmap, GraphPad Prism v10.0, and Adobe Illustrator 2024; schematics were created with BioRender.com.

## RESULTS

### Clinical outcomes following HMA‐venetoclax therapy in AML

A total of 132 patients diagnosed with AML, including both newly diagnosed and relapsed/refractory cases, and treated with HMA in combination with venetoclax (HMA‐Ven), were included for outcome assessment. Among this cohort, 10 sequential patients were selected for single‐cell RNA sequencing (scRNAseq) to investigate biological features associated with treatment response (Figure 1A). Clinical characteristics of the entire cohort are summarized in Supporting Information S1: Tables 1 and 2.

**Figure 1:**
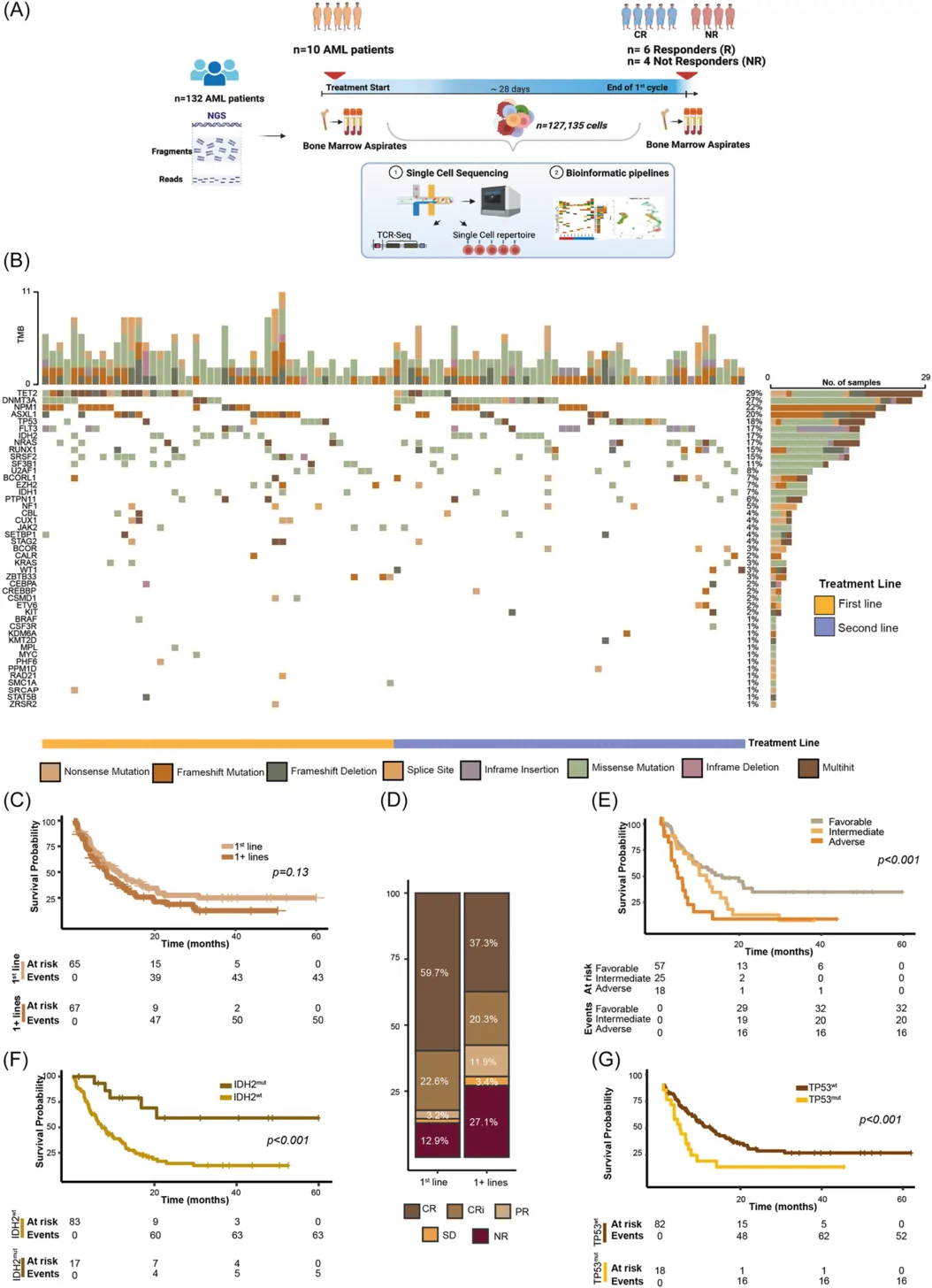
**TP53 mutational status correlates with inferior progression‐free survival across treatment lines and modalities**. **(A)** Schematic overview of the study design. Targeted NGS profiling was performed on 99 AML patients treated with HMA‐Venetoclax. A subset of 10 newly diagnosed patients underwent paired single‐cell RNA sequencing and T cell receptor (TCR) profiling at diagnosis and after one cycle of therapy. **(B)** Distribution of genetic mutations across the cohort, stratified by treatment line (first vs. later lines). Mutation types include nonsense, frameshift, in frame, splice site, missense, and multi‐hit variants. NGS diagnostic data were available in 99 patients **(C)** Kaplan–Meier curves comparing progression‐free survival (PFS) between patients treated in either first or later lines of therapy (P = 0.13). **(D)** Remission rates in patients treated in either first or later lines of therapy (CR, complete response; CRi, complete response with incomplete count recovery; NR, no response; PR, partial response; SD, stable disease). **(E)** Survival probability stratified according to ELN2024 prognostic risk. **(F)** Survival comparison between IDH2 WT and mutant patients treated with HMA‐Venetoclax (P < 0.001). **(G)** Survival comparison between TP53 WT and mutant patients treated with HMA‐Venetoclax (P < 0.001).

Next‐generation sequencing (NGS)‐based molecular profiling was available for 99 out of 132 patients (75.7%). The most frequently mutated genes included TET2 (29%), DNMT3A (27%), NPM1 (22%), ASXL1 (20%), and TP53, FLT3, IDH2, and NRAS (each 17%) (Figure 1B).

Of the total cohort, *n* = 65 patients (49.2%) received HMA‐Venetoclax as first‐line therapy, while *n* = 67 patients (50.8%) were treated in subsequent lines. At the time of survival analysis update, the median follow‐up was 17.06 months. No statistically significant difference in overall survival (OS) was observed between patients treated in first or later lines of treatment (Figure 1C).

Composite complete remission rates (CR + CRi) were significantly higher in patients receiving HMA‐Venetoclax as first‐line therapy (82.3% vs. 57.6%, P < 0.01; Figure 1D), although response rates in the relapsed/refractory group remained consistent with those reported in the VIALE‐A trial.^8^, ^9^

A genomic risk stratification based on the mutational status of NPM1, IDH1, IDH2, FLT3‐ITD, N/KRAS, and TP53 as per ELN2024 consensus was applied and retained prognostic relevance in the whole cohort but performed better in patients treated in first line, consistent with ELN2024 (Figure 1E and Supporting Information S1: Figure 2A). OS—as measured from the start of HMA‐Venetoclax treatment—was significantly different between favorable and adverse risk groups (Log‐rank P < 0.001), with the intermediate group trending toward significance when compared to favorable and adverse risk groups (P = 0.05). Patients harboring IDH2 mutations had not yet reached median OS, suggesting favorable outcomes, while those with TP53 mutations exhibited the poorest prognosis with a median OS of only 4.35 months (Figure 1F). Patients with NPM1 mutation, whether treated in first or later lines, did not experience any survival benefit in our cohort, possibly given the relatively small sample size (*n* = 22). Analogously, patients with IDH1 mutation did not display any OS benefit, consistent with ELN2024 classification, as favorable outcomes are observed in the setting of ivosidenib exposure only (Supporting Information S1: Figure 2B). Patients who had received alloSCT at any point during treatment experienced the largest OS benefit, stressing the potential of this treatment both as bridge to transplant and in the post‐SCT relapse setting (Supporting Information S1: Figure 2C). Taken together, these real‐world data confirm that HMA‐Venetoclax is effective in both treatment‐naive and previously treated AML patients, with CR+CRi rates approaching 60% even in the latter group. Moreover, the ELN2024 classification, although designed for front‐line therapy, maintained relevant prognostic significance in this heterogeneous cohort. Finally, NGS‐based profiling confirmed the positive prognostic impact of IDH2 mutations and the adverse outcomes associated with TP53 mutations.

### Single‐cell profiling of AML reveals response‐associated shifts in cellular frequencies after HMA‐venetoclax treatment

We performed single‐cell RNA sequencing (scRNA‐seq) on a total of 127,135 high‐quality BM cells from 10 patients with newly diagnosed AML treated with HMA‐Venetoclax. Samples were collected at diagnosis (TP1, *n* = 58,643) and following completion of the first treatment cycle (TP2, *n* = 68,492). Clinical trajectories and molecular features of these patients are summarized in Figure 2A.

**Figure 2:**
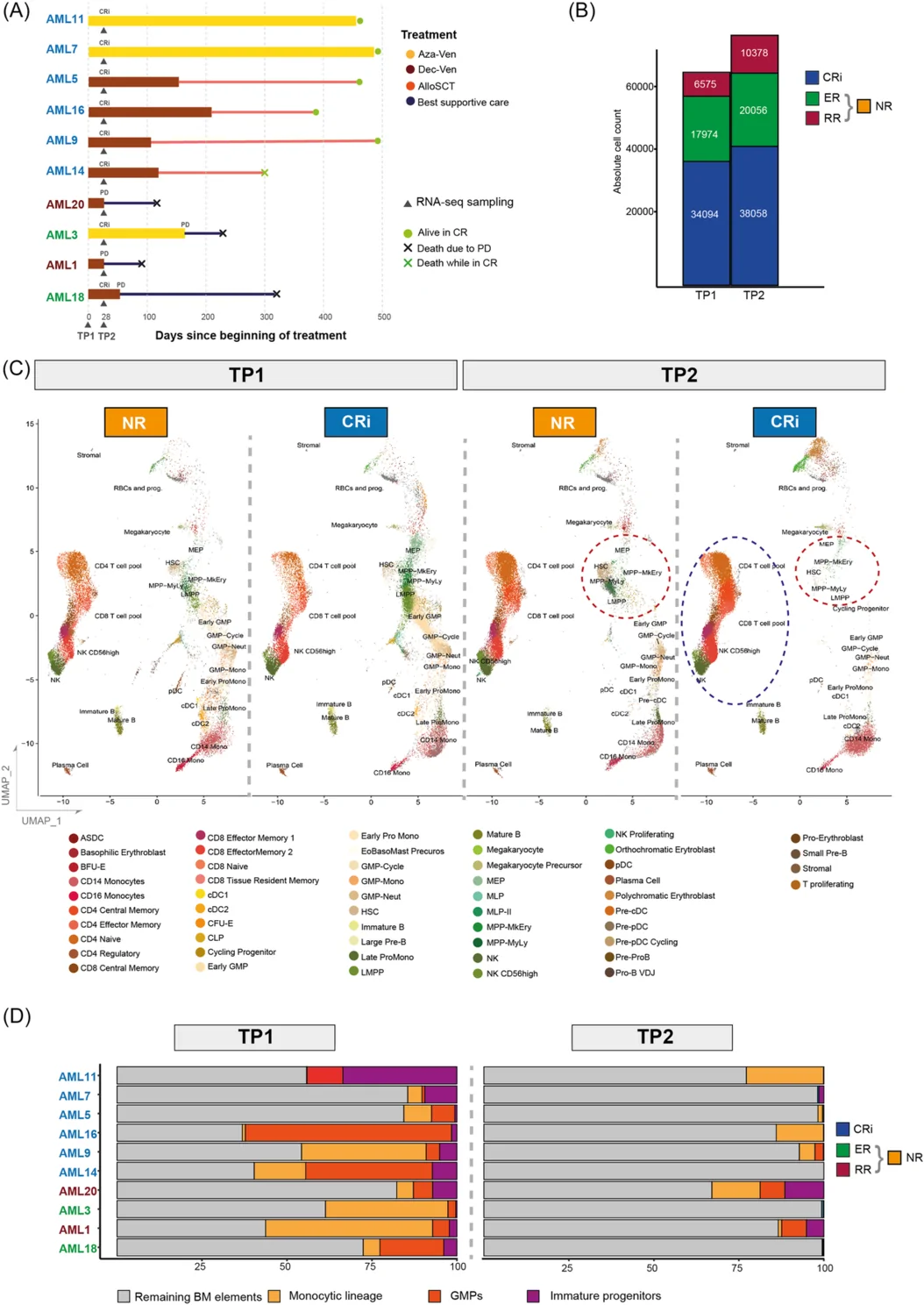
**Single‐cell profiling reveals response‐associated shifts in cellular composition and leukemic phenotypes following HMA‐Venetoclax therapy**. **(A)** Treatment timelines for AML patients included in the single‐cell RNA‐seq cohort. Bars indicate therapy received (Azacitidine/Decitabine ± Venetoclax), with symbols denoting RNA‐seq sampling, death in complete remission (CR), or death outside CR. **(B)** Stacked bar plots showing the distribution of cells at diagnosis (TP1) and post‐cycle one (TP2), stratified by response group (CRI vs. NR and RR). **(C)** UMAP projections of annotated hematopoietic and immune cell types across CRI and NR/RR groups at TP1 and TP2. Responders show posttreatment expansion of CD8^+^ T cells and early progenitors (erythroid/megakaryocytic), while nonresponders retain persistent T‐cell infiltration and limited hematopoietic recovery. **(D)** Cellular myeloid composition across AML samples at TP1 and TP2. Bar plots display proportions of monocyte lineage, GMPs, and myeloid cells, highlighting dynamic shifts in responders and phenotypic persistence in refractory cases. Red and blue circles highlight the difference in immature myeloid and T cell composition, respectively, observed between NR and Cri patients after treatment exposure (TP2).

The median cell yield per sample was 4620 (range, 1127–18,083). Responders contributed 34,094 and 38,058 cells at TP1 and TP2, respectively, while patients who achieved a short‐lived response (ER, duration of less than 4 cycles of treatment) contributed 6575 and 10,378 cells. Patients with refractory disease (RR) had 17,974 and 20,056 cells at TP1 and TP2, respectively (Figure 2A,B and Supporting Information S1: Figure 1).

No significant differences in cell recovery were observed between patients achieving durable response versus patients failing treatment (P > 0.5, Wilcoxon test; Supporting Information S1: Figure 3). Patients received either azacitidine (*n* = 7) or decitabine (*n* = 3) in combination with Venetoclax, for a median of four cycles (range, 2–14). At TP2, 8 out of 10 patients (80%) achieved complete remission (CR or CRi). Four patients proceeded to allogeneic hematopoietic stem cell transplantation. Clinical characteristics of the single‐cell cohort are reported in Supporting Information S1: Table 2. Patients failing treatment were enriched for TP53 mutations (1/4) and NRAS (2/4), while the remaining patient progressed from an NPM1‐DNMT3A‐TET2 mutated AML with a missense, noncanonical FLT3 mutation.

Cell identities were annotated using a reference Bone Marrow Map, enabling classification of 92% of cells across the hematopoietic hierarchy, from hematopoietic stem cells (HSCs) and multipotent progenitors (MPPs) to granulocyte‐monocyte progenitors (GMPs) and mature monocytes (CD14^+^/CD16^+^) (Figure 2C). At baseline, responders were enriched in GMP‐ and MPP‐like compartments, whereas nonresponders exhibited increased T‐cell fractions, particularly CD4^+^ and CD8^+^ subsets. At TP2, responders showed a marked expansion of CD4^+^ and CD8^+^ T‐cells and early erythroid/megakaryocytic progenitors, indicative of nascent hematopoietic recovery. In contrast, nonresponders exhibited persistent T‐cell infiltration from diagnosis. Notably, the emergence of red blood cell progenitor populations in responders, despite an incomplete hematologic recovery, suggests treatment‐induced activation of hematopoiesis (Figure 2C and Supporting Information S1: Figure 4).

Of the 10 patients subjected to sc‐RNA‐Seq, 7 had abnormal karyotypic analysis (Supporting Information S1: Figure 5). In all these cases, CNV information inference by means of the Numbat algorithm tracked the expected cytogenetic abnormalities mostly to cells of myeloid derivation (Supporting Information S1: Figure 5A,B), supporting their nature as blast cells. In the remaining cases (3/10), no direct proof of clonality matching CNAs with the known diagnostic karyotype was available.

AML cases were then further stratified at diagnosis based on the predominant leukemic population into three phenotypic classes: immature (HSC/MPP‐dominant), intermediate (GMP‐dominant), and mature (monocytic‐dominant). Four patients exhibited mature monocytic profiles, three had intermediate GMP‐dominant, and three displayed an immature HSC/MPP phenotype. These designations were corroborated by clustering analyses: monocytic clusters were enriched for FGR, CD14, MNDA, and S100A8/9; GMP‐dominant clusters expressed MPO and PRSS57, while immature clusters were characterized by stem‐like transcripts such as CD34 and HOPX (Supporting Information S1: Figure 6).

Among nonresponders, two patients presented with monocytic AML, one with an intermediate GMP profile, and one with an immature HSC/MPP‐dominant phenotype. Post‐treatment, refractory cases displayed lineage shifts or persistence of immature transcriptional states. In one RAS‐mutated patient initially classified as monocytic, scRNA‐seq at TP2 revealed a shift to an immature phenotype, suggestive of lineage regression as a potential resistance mechanism (Figure 2D). RNA velocity analysis further confirmed a phenotype switch at TP2 characterized by cells with immature features, indicating dynamic regression from a monocytic to a GMP/MPP‐like state in this patient (Supporting Information S1: Figure 7A,B). Proof of clonal relationship of these cell types across time is provided by the shared trisomy 8 and deletion 17 tracked through the Numbat algorithm (Supporting Information S1: Figure 5A,B, AML1 TP1 and TP2). A second refractory case, marked by a TP53 mutation and an immature phenotype at diagnosis, exhibited persistence of immature HSC/MPP‐like populations following therapy, consistent with survival of a treatment‐refractory leukemic clone (Figure 2D).

### Transcriptomic signatures of treatment response and refractory disease in AML patients treated with HMA‐venetoclax

To delineate therapy‐related molecular changes in the BM microenvironment, we performed gene set enrichment analysis (GSEA) on 127,135 cells RNA expression data from 10 AML patients, comparing samples collected at diagnosis (TP1) and after one cycle of HMA‐Venetoclax treatment (TP2). Comparative transcriptomic analyses revealed divergent activation of inflammatory and stromal signaling pathways that distinguished responders from nonresponders (Figure 3A–C).

**Figure 3:**
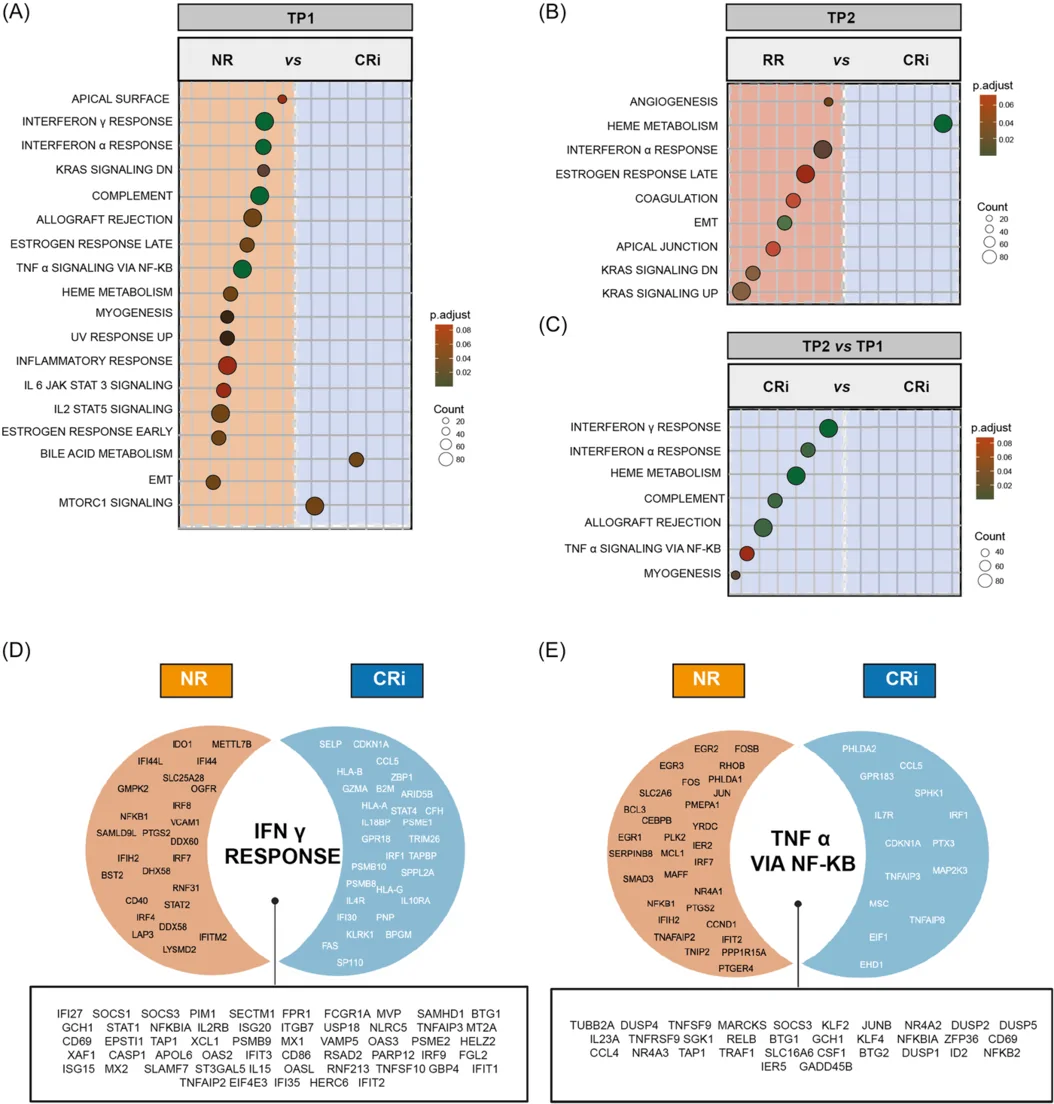
**Transcriptomic profiling reveals divergent inflammatory and stromal signaling in AML patients treated with HMA‐Venetoclax**. **(A)** Gene set enrichment analysis (GSEA) comparing nonresponders (NR) and patients achieving complete remission with incomplete count recovery (CRi) at baseline (TP1), highlighting upregulation of IFNγ, TNFα/NF‐κB, and EMT‐related pathways in NR. **(B)** GSEA comparing refractory cases (RR) and CRi at TP2, showing persistent inflammatory signaling in RR. **(C)** Longitudinal GSEA in CRi patients between TP1 and TP2, indicating treatment‐induced activation of IFN and antigen presentation pathways. **(D)** Venn diagram of leading‐edge genes from the IFNγ response gene set in NRvsCRi at TP1 versus CRi TP1vsTP2. **(E)** Venn diagram of leading‐edge genes from the TNFa via NFKB response gene set in NRvsCRi at TP1 versus CRi TP1vsTP2. Shared genes are listed below each diagram. Dot size reflects gene count; color scale indicates adjusted p‐value (Benjamini‐Hochberg correction).

At baseline, patients failing treatment (NR including ER + RR) exhibited significant upregulation of IFN and TNFα/NF‐κB signaling pathways at FDR < 0.05 (Figure 3A), suggestive of a pre‐existing inflammatory and potentially immunosuppressive microenvironment. The same modules were found upregulated at TP2 when comparing RR to patients achieving CRi (Figure 3B). In contrast, these same pathways were activated only after treatment in responders (Figure 3C), indicating distinct temporal dynamics of immune activation. Longitudinal profiling of nonresponders revealed progressive upregulation of IFN‐related genes, hypoxia signatures, and epithelial to mesenchymal transition (EMT) programs at TP2 (Supporting Information S1: Figure 8A–D), consistent with a microenvironment primed for resistance.

IFN and TNF gene sets are quite large, so their enrichment might stem from upregulation of different genes in different contexts and not have the same biological meaning. Further dissection of the IFN‐γ response gene set identified 25 and 29 genes private to nonresponders and responders, respectively (Figure 3D). Inflammatory pathways were found to be activated in all 4 patients failing treatment (ER + RR) when compared individually to the pool of patients achieving durable response. In responders, IFN‐γ enrichment was driven by upregulation of MHC class I molecules (HLA‐A, HLA‐B, HLA‐C, HLA‐G), inflammatory chemokines (CCL5), and transcriptional regulators such as STAT4, which mediate Th1 and Th17 differentiation, supporting the emergence of an immune‐stimulatory milieu conducive to leukemic clearance. Conversely, nonresponders demonstrated a tolerogenic and pro‐leukemic signature, marked by expression of IDO1, METTL7B, and VCAM1 (Figure 3D). Single‐cell resolution localized IDO1 expression primarily to conventional dendritic cells (cDC1) at TP1, although recovery of this population was limited (≥50 cells) and detected in only one case with progressive disease (Supporting Information S1: Figure 9A,B). TNFα/NF‐κB signaling was similarly enriched in nonresponders at diagnosis and in responders posttreatment; however, functional divergence was evident in the leading‐edge genes. In nonresponders, this signature included MCL1, FOS, FOSB, and RHOB (genes implicated in leukemic survival, resistance, and invasiveness), particularly in myeloid cells with immature (HSC‐like) and monocytic differentiation, while in responders, enrichment reflected posttreatment inflammatory activation (Supporting Information S1: Figure 10).

These data show that constitutive activation of IFN and NF‐κB signaling in nonresponders reflects pretreatment immune dysfunction and a BM niche supportive of therapeutic resistance. In contrast, the inducible activation of these pathways in responders may facilitate immune‐mediated antileukemic responses, confirming that more granular dissection of large gene sets can reveal marked transcriptomic divergence and better explain the translational impact of this analysis. Furthermore, scRNA‐seq analysis allows investigation of the actual cell types involved in IFN signaling. In particular, expression of the intracellular IFN effector STAT1 was found to be upregulated across most cell types in ER and RR patients at TP1, including myeloid progenitors, cells of monocytic derivation, and immune cells (Supporting Information S1: Figure 11A,B). This difference appeared, again, less marked and not statistically different in cells of monocytic derivation at TP2 when compared to cells belonging to patients achieving CR. Despite the limited size of our cohort, these data point toward a dynamic IFN activity governing treatment response.

EMT‐related programs were recurrently upregulated in nonresponders (FDR < 0.05), most notably in the patient harboring a TP53 mutation. EMT gene expression, including PRSS2, LGALS1, as well as BASP1, was maintained across timepoints, consistent with ongoing stromal remodeling. Finally, sustained activation of RAS signaling was observed in two patients—both failing to achieve sustained response—at both TP1 and TP2 (FDR < 0.05), reinforcing the contribution of oncogenic RAS to primary Venetoclax resistance^16^ (Supporting Information S1: Figure 12A,B).

### Transcriptional profiling reveals inflammatory rewiring and stem‐like programs associated with venetoclax resistance in AML blasts

To investigate mechanisms of resistance to HMA‐venetoclax therapy in specific cell subsets in AML, we analyzed the pathways described above within different myeloid maturation states by performing integrative transcriptomic profiling, combining single‐cell RNA sequencing of myeloid compartments with GSEA of matched transcriptomes according to response. Across differentiation states, nonresponders again exhibited a pervasive enrichment of inflammatory gene programs, including IFN and TNFα/NF‐κB signaling, already present at baseline. Notably, these inflammatory signatures were independent of our predefined maturation state and appeared as a core feature of resistance (Figure 4A–C).

**Figure 4:**
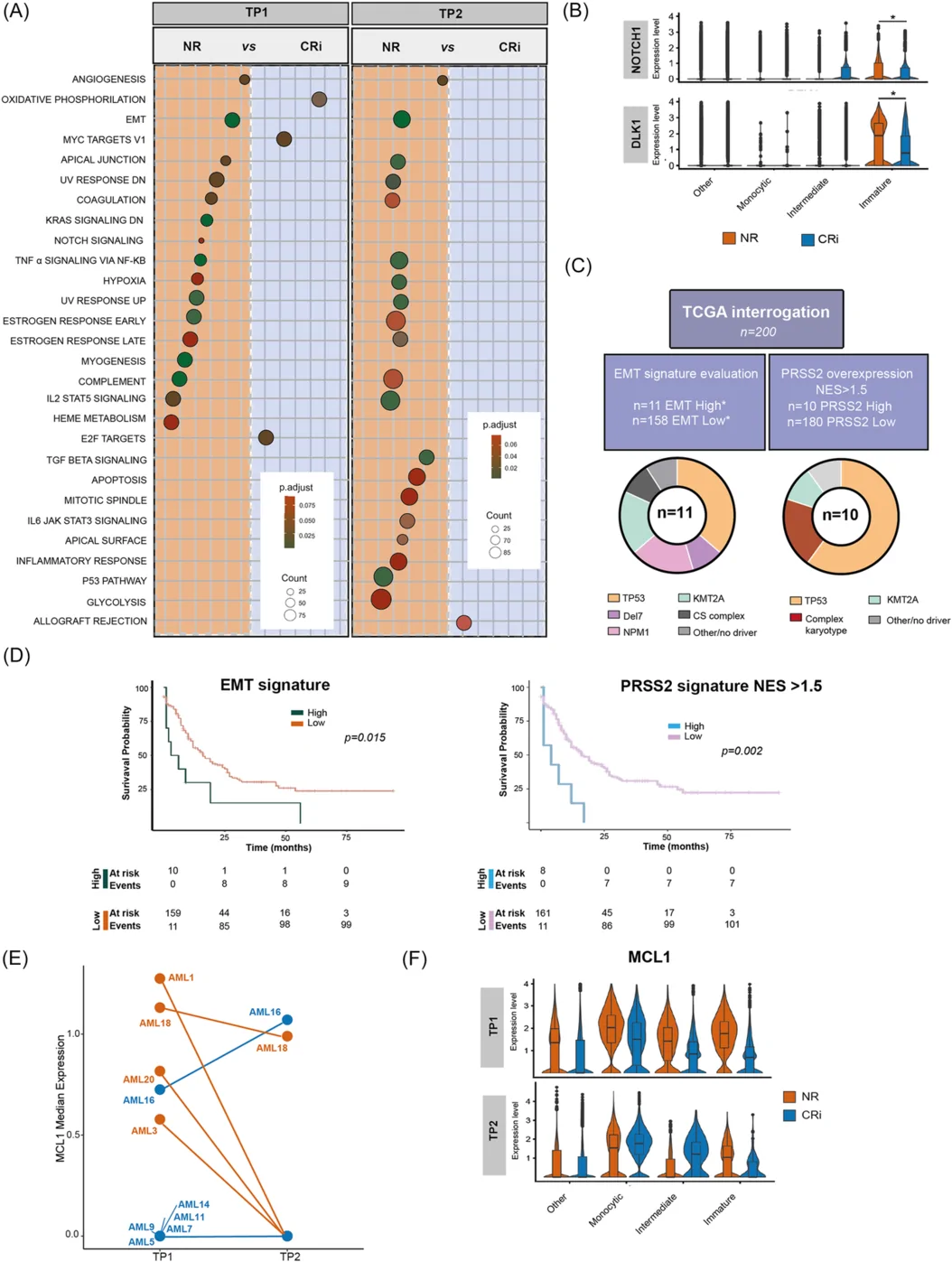
**Transcriptional profiling reveals inflammatory rewiring, NOTCH signaling, and apoptotic remodeling associated with venetoclax resistance in AML blasts**. **(A)** Gene set enrichment analysis (GSEA) comparing nonresponders (NR) and responders (CRi) at baseline (TP1) and post‐treatment (TP2) within myeloid blast populations. **(B)** Violin plots showing higher expression of *NOTCH1* and *DLK1* in immature myeloid progenitors in NR compared to CRi (P < 0.01). **(C)** Analysis of the TCGA AML cohort (*n* = 200) evaluating mutational distributions in patients stratified by EMT signature score (high vs. low) and *PRSS2* expression (NES > 1.5). **(D)** Kaplan–Meier curves from the TCGA dataset demonstrating significantly shorter overall survival in patients with high EMT signature (P = 0.015) and *PRSS2* overexpression (P = 0.002). **(E)** Longitudinal tracking of median *MCL1* expression across individual patients at TP1 and TP2, highlighting persistence or elevation in nonresponding cases. **(F)** Violin plots depicting *MCL1* transcript levels across distinct myeloid maturation compartments (Other, Monocytic, Intermediate, Immature) at TP1 and TP2 stratified by response (NR vs. CRi).

Developmental pathway analysis revealed that NOTCH signaling was selectively active in immature myeloid cells, including HSCs and MPPs, and was enriched in TP53‐mutant AML. Canonical (*HES1, NOTCH1, JAG1*) and noncanonical (*DLK1*) NOTCH components were upregulated in two refractory cases (Figure 4A,B). EMT programs were activated in immature and intermediate myeloid subsets but were absent in terminal monocytic cells. EMT‐related genes (*PRSS2, LGALS1, BASP1*) were particularly enriched in the TP53‐mutant case and intensified after therapy, alongside upregulation of TGFB signaling. These findings suggest establishment of an autocrine EMT loop in immature leukemic cells under treatment pressure (Figure 4A). To validate these findings in a larger cohort, we mined data from the TCGA AML dataset (*n* = 200 AML patients). Here, EMT‐high patients (ssGSEA score >0.39, log‐rank maximization) had significantly shorter OS (mOS 4.5 vs. 16.06 months, P‐value = 0.01). *PRSS2* overexpression (>1.5 normalized expression) also marked a high‐risk subgroup enriched for TP53 and other adverse mutations, with EMT signature enrichment further stratifying outcomes within TP53‐mutant cases (Figure 4C–D and Supporting Information S1: Figure 13). Multivariate analysis, however, identified TP53 mutation as the most determinant factor for adverse prognosis regardless of EMT score levels (Supporting Information S1: Figure 14).

In our scRNA‐seq cohort of 10 patients, we also identified persistent RAS pathway activation across timepoints in two patients who failed to achieve long‐term remission, with expression observed in both monocytic and progenitor compartments. While MCL1 levels were higher at baseline, median MCL1 levels measured on the whole BM cellularity were comparable across response groups after treatment start (Figure 4E). However, all 4 ER and RR patients had elevated *MCL1* expression at TP2 selectively in immature blasts and monocytic cells, suggesting MCL1 upregulation as a mechanism of resistance only restricted to the myeloid compartment. *BCL2A1* was also upregulated post‐treatment in ER and RR, particularly in the patient with a TP53 mutation, while responders showed a tendency toward increased *BCL2L1*, pointing to divergent apoptotic remodeling (Figure 4F and Supporting Information S1: Figure 15). Pro‐apoptotic genes including *BAK* and BH3‐only proteins did not track treatment response. Taken together, these data define a resistance program in AML characterized by constitutive inflammatory signaling, NOTCH‐driven stemness, EMT‐mediated lineage plasticity, and apoptotic evasion centered on *MCL1*, *BCL2A1*, and IAPs. In contrast, responders exhibit treatment‐induced immune activation and apoptotic priming. These findings underscore the interplay between cell‐intrinsic factors in shaping the response to venetoclax‐based therapy and suggest candidate pathways for therapeutic targeting.

### CD4^+^ T cell phenotypes and clonal remodeling stratify response to venetoclax‐based therapy

To explore the contribution of T‐cells to response heterogeneity in AML treated with HMA–Venetoclax, we analyzed *n* = 41,755 single T‐cells with paired TCR sequencing from longitudinal BM samples (*n* = 10 patients). CD4^+^ T‐cells (*n* = 24,721) were classified into functional subsets using the ProjectTILs framework. At baseline (TP1), ER and RR patients displayed a significantly higher proportion of CD4^+^ T‐cells compared to responders (16.1% vs. 7.9% of total marrow cells, Chi‐square P < 0.01), a pattern reversed posttreatment (TP2), with CD4^+^ cells expanding in responders (29.5% vs. 22.0%, Chi‐Square P < 0.01; Figure 5A). Despite naïve‐like cells dominating the CD4 compartment in both groups, responders demonstrated a marked increase in small‐frequency clonotypes over time, consistent with TCR diversification and immune activation. In contrast, ER + RR patients showed minimal clonal remodeling (Figure 5B). Unsupervised clustering identified five distinct CD4 transcriptional states. Cluster 1, enriched in ER + RR at both timepoints (Chi Square test, P < 0.01), expressed regulatory and stress‐associated genes (*FOS, NFKB1, USP51, BACH2*). Cluster 2, predominant in responders, showed cytotoxic gene expression (*GZMA, GZMK, ZNF683*), while Cluster 3 included naïve T‐cells (Chi‐Square test, P < 0.01). Cluster 4 mapped to regulatory T‐cells (*FOXP3, RTKN2*), and Cluster 5, observed exclusively in ER + RR patients at TP2, expressed *IL22*, suggesting a tumor‐supportive inflammatory phenotype (Figure 5C,D).

**Figure 5:**
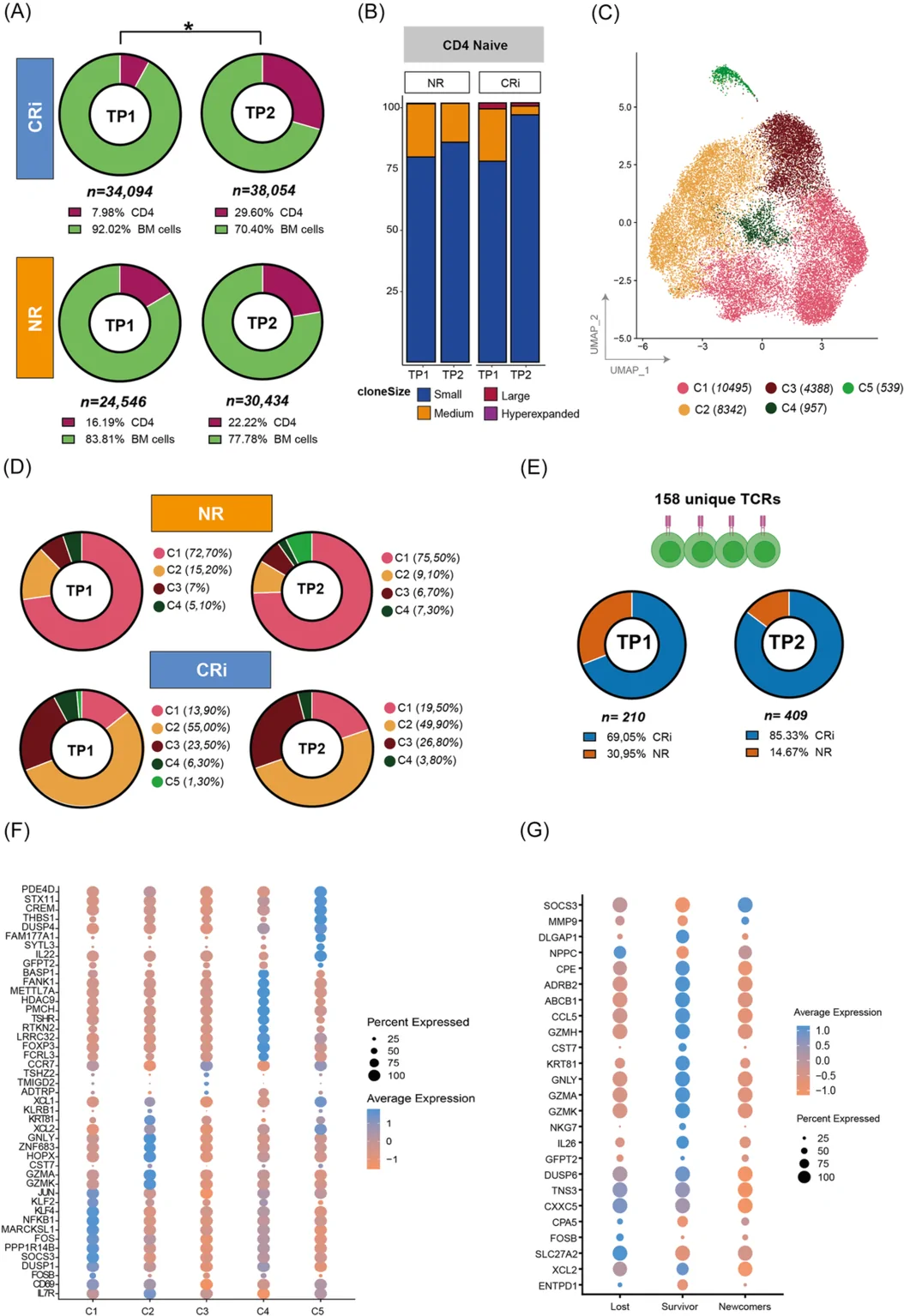
**CD4^+^ T cell remodeling and clonal dynamics stratify response to HMA‐Venetoclax in AML**. **(A)** Proportions of CD4^+^ T‐cells and bone marrow (BM) cells at TP1 and TP2 in CRi and NR patients. **(B)** Clone size distribution of CD4^+^ naïve T‐cells across response groups and timepoints. **(C)** UMAP clustering of CD4^+^ T‐cells identifies five transcriptional states (C1–C5). **(D)** Cluster distribution in CRi and NR patients at TP1 and TP2, showing enrichment of cytotoxic subsets in CRi and regulatory/exhausted profiles in NR. **(E)** Longitudinal tracking of 158 unique CD4^+^ TCRs reveals increased persistence and expansion in CRi. **(F)** Dot plot of gene expression across clusters C1–C5, highlighting functional divergence. **(G)** Gene expression profiles of lost, persistent (survivor), and newly emerging CD4^+^ clonotypes, showing cytotoxic remodeling in CRi and immunosuppressive features in NR. Dot size reflects percent expression; color scale indicates average expression.

Longitudinal tracking of clonotypes revealed a total of 158 persistent CD4 TCRs, primarily in responders. These persisting clones upregulated cytotoxic mediators (*GZMA, GZMH, NKG7*), indicative of sustained anti‐leukemic function. In contrast, clonotypes lost over time expressed exhaustion‐related and suppressive transcripts (*FOSB, CD39, SLC27A2*), while newly emerging clones exhibited a mixed program involving immune regulation and tissue remodeling (*CD80, MMP9, OSM, SOCS3*; Figure 5E–G). Expansion or persistence of CD4 T cells with persisting TCRs was observed in 3/6 patients achieving CR and in 0/4 of ER + RR patients (Supporting Information S1: Table 3), showing that, even in our small cohort, this initial observation did not result from a disproportionate contribution of a single patient.

These findings suggest that response to Venetoclax‐based therapy is associated with dynamic reshaping of the CD4 compartment, presence of cytotoxic CD4 subsets, and their persistence across timepoints, while treatment failure is characterized by stable, immunosuppressive profiles and pro‐tumorigenic phenotypes.

### Expansion and cytotoxic remodeling of CD8^+^ T cells associate with favorable outcome

CD8^+^ T cells also demonstrated marked differences between responders and nonresponders. At diagnosis, ER and RR patients had a higher frequency of CD8^+^ T cells (13.6% vs. 9.6% of marrow cells), which declined posttreatment (11.3%), whereas responders exhibited robust CD8^+^ expansion (from 9.6% to 18.2%, P < 0.001; Figure 6A). Notably, only responders developed hyperexpanded CD8^+^ clones within the terminal effector (TEMRA) subset, particularly in long‐term survivors. TCR analysis revealed static diversity in ER + RR patients, while responders showed increased frequencies of small clonotypes across CD8^+^ subsets after treatment (Figure 6B). Functionally, TEMRA cells from responders expressed cytotoxic markers (ZNF683, PCBP2), whereas those from nonresponders were enriched in dysfunctional and inflammatory genes (JUND, FOSB, DUSP2). Effector memory (EM) CD8^+^ cells in ER + RR patients expressed exhaustion‐related transcripts (LAG3, NEAT1, IFITM2), while central memory cells displayed increased inhibitory signals (KLRD1, JUN). In contrast, the responders' EM subsets showed higher expression of CXCR4, potentially supporting homing and activation (Figure 6C). Longitudinal tracking identified 607 persistent CD8^+^ clonotypes, with selective expansion in responders (from 1810 to 3398 cells posttreatment), predominantly within the TEMRA compartment. These persisting clones in responders expressed cytotoxic and activation markers (ZNF683, ITM2A, ITGAM), while nonresponder clones upregulated inflammatory and dysfunctional genes (DUSP2, JUN, FOS; Figure 6D,E). This effect was driven mainly by 4 out of 6 patients achieving response, with 3 out of 4 also experiencing CD4 T cell expansion/persistence (Supporting Information S1: Table 4). Only one patient failing treatment experienced unique TCR CD8 T cell expansion. Checkpoint profiling revealed limited PD‐1, CTLA‐4, and TIM‐3 expression. However, LAG‐3 was persistently expressed in ~25% of CD8^+^ T cells in nonresponders and remained elevated posttreatment, particularly in EM subsets, suggesting a phenotype consistent with an exhaustion‐like state rather than demonstrated functional exhaustion (Supporting Information S1: Figure 16A–D). Together, these results demonstrate that clinical response to Venetoclax is accompanied by CD8^+^ T cell clonal diversification, cytotoxic reprogramming, and sustained effector expansion. In contrast, nonresponders exhibit blunted remodeling of TME, persistent LAG‐3 expression, and enrichment of inflammatory‐exhausted phenotypes, implicating immune dysfunction as a potential barrier to durable remission.

**Figure 6:**
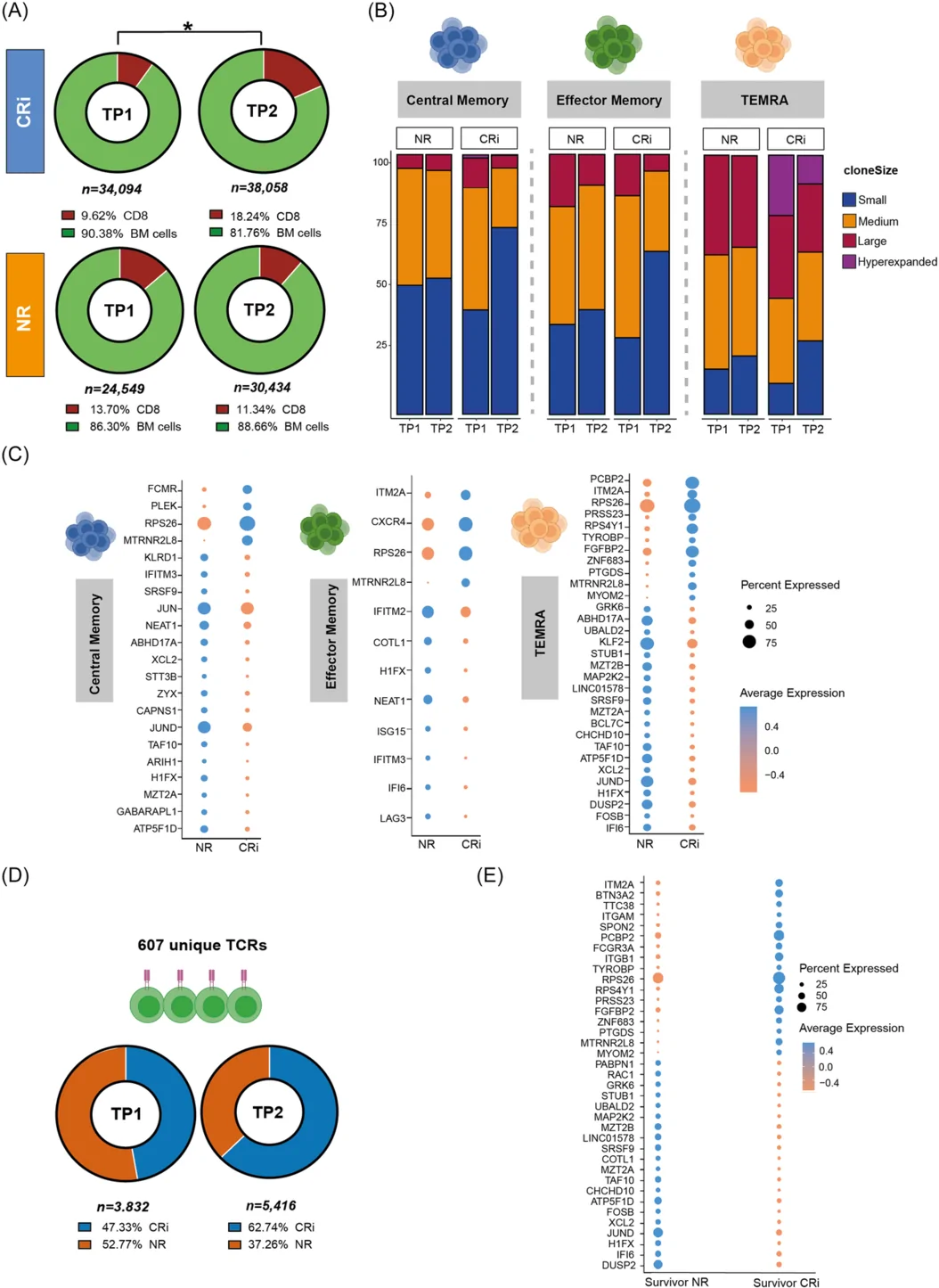
**Expansion and cytotoxic remodeling of CD8^+^ T‐cells associate with favorable outcome**. **(A)** CD8^+^ T cell frequency at diagnosis (TP1) and posttreatment (TP2) in responders and nonresponders. Responders show robust expansion posttreatment, while nonresponders exhibit a decline. **(B)** Distribution of CD8^+^ T cell subsets and clonotype diversity across response groups and timepoints. Only responders develop hyperexpanded clones within the TEMRA compartment. **(C)** Dot plots showing gene expression profiles of Central Memory, Effector Memory, and TEMRA cells in responders and nonresponders. **(D)** Longitudinal tracking of CD8^+^ clonotypes reveals selective expansion in responders, predominantly within TEMRA. **(E)** Dot plots showing gene expression profiles.

## DISCUSSION

Using integrated DNA sequencing along with single‐cell RNA and TCR sequencing, we characterized the clonal population and BM immune microenvironment of AML patients treated with HMA‐Venetoclax, revealing key molecular and cellular correlates of therapeutic response. Our findings indicate that resistance to treatment is closely associated with immature myeloid phenotypes driven by NOTCH and RAS signaling, particularly in cases harboring TP53 or NRAS mutations. Although pleomorphic in activity, NOTCH1 may be implicated in TME interactions mediating treatment resistance and niche remodeling.^17^, ^18^ These immature states often persisted or emerged after therapy, consistent with recent evidence showing impaired differentiation in NRAS‐mutant AML resistant to Venetoclax.^16^, ^18^ These findings are consistent with recent single‐cell studies of venetoclax‐treated AML, which similarly reported monocytic clone persistence via MCL1/glycolytic and CXCL12‐CXCR4‐driven resistance,^19^ BCL2‐pathway remodeling in resistant clones,^20^ and MYC‐high HSPC expansion with impaired CD4 + T‐cell immunity in nonresponders.^19^, ^20^, ^21^ While monocytic differentiation may be associated with treatment resistance, refractory patients with monocytic disease exhibited a shift toward an immature phenotype upon treatment exposure. We acknowledge that the limited size of the single‐cell cohort, and particularly the small number of nonresponders, constrains the generalizability of these findings; several signatures described above (e.g., RAS and EMT activity) are driven by individual mutation‐specific cases and should therefore be considered hypothesis‐generating pending validation in larger, independent cohorts.

At baseline, nonresponders exhibited heightened inflammatory signaling, including IFN and NF‐κB pathways, fostering a suppressive BM milieu unfavorable to effective immune activation. In contrast, responders showed inducible IFN responses only after blast clearance, alongside increased expression of MHC class I and immunostimulatory genes such as CCL5 and FAS. Notably, chronic IFN signaling in nonresponders correlated with tolerogenic markers, i.e., IDO1, METTL7B, and VCAM1, especially within dendritic cell populations, suggesting an immunosuppressive niche that may contribute to T‐cell dysfunction via disrupted antigen presentation and toxic metabolite production.^22^, ^23^ STAT1 expression appeared to be consistently higher across multiple cell types in patients failing treatment, particularly at TP1, while this difference was mitigated after treatment exposure, especially in cells of monocytic derivation. This again hints toward a potential deleterious effect of chronic IFN stimulation of the TME on treatment outcomes. Functional validation in larger cohorts will be required to confirm these findings.

Overall, inflammatory signals were prominent both in responders and in nonresponders, albeit at different timepoints and with different functional implications. Analysis of the T‐cell repertoire further substantiated this apparent conundrum. Responders demonstrated dynamic clonal remodeling and expansion, particularly within cytotoxic CD4 and CD8 TEMRA subsets, with increased TCR diversity and persistence‐expansion of effector clones expressing markers, including ZNF683, GZMA, and PCBP2, indicative of ongoing immune surveillance. Conversely, nonresponders harbored static repertoires enriched in T‐cells expressing inflammatory and exhaustion‐associated genes (FOS, JUND, DUSP1/2), predominantly in the CD8 compartment. These surviving CD8 T‐cells exhibited transcriptional profiles suggestive of chronic cytokine exposure rather than effective cytotoxicity, reflecting dysfunctional or misdirected immune responses, a pattern aligned with prior AML studies, particularly in cohorts enriched for chromatin‐spliceosome mutations.^24^, ^25^ Longitudinal tracking of unique TCRs revealed selective expansion of pre‐existing cytotoxic CD4 clones in responders, implying immune augmentation post‐treatment, a phenomenon previously described in solid tumors.^26^, ^27^ Similarly, responders showed preferential selection of cytotoxic CD8 T cell clones. Importantly, treatment with HMA‐Venetoclax did not diminish T cell abundance; rather, responders exhibited a significant increase in CD8 T‐cells following therapy, suggesting neutral or even beneficial effects on T cell populations, consistent with recent findings proposing HMA‐Venetoclax as a platform to enhance subsequent immunotherapies.^28^

Expression of conventional exhaustion markers such as PD‐1, CTLA‐4, and TIM‐3 was generally low across patients, with PD‐1 detected in only ~10% of T‐cells at diagnosis and downregulated post‐treatment, likely reflecting depletion of pre‐exhausted populations. This may explain the limited success of checkpoint inhibitors targeting these pathways in AML thus far.^29^, ^30^ By contrast, LAG3 was the most consistently expressed checkpoint receptor in nonresponder patients, persisting across timepoints and T cell subsets, potentially implicating a dysfunctional T‐cell population. Functional studies will be needed to provide further insight into this initial observation. Indeed, further exploration of the role of LAG3 in AML is warranted, given also the availability of targeted inhibitors.^31^ Together, our data delineate an intricate interplay between leukemic genotype, myeloid differentiation states, inflammatory signaling, and T cell functional dynamics in determining HMA‐Venetoclax response. Resistance is characterized by a convergence of immature myeloid phenotypes, chronic inflammation, and rigid, dysfunctional T cell responses marked by immunoregulatory skewing. In contrast, responders display effective T cell engagement, cytotoxic persistence, and adaptive repertoire remodeling, consistent with an active immune role in disease clearance that can arise rapidly after treatment initiation.

One limitation of our study is its limited sample cohort. However, our initial observations underscore the potential of combining HMA‐Venetoclax with interventions aimed at restoring T cell fitness or alleviating immune suppression within the leukemic niche to sustain and consolidate clinical remission in AML.

## AUTHOR CONTRIBUTIONS

**Alfredo Marchetti**: Conceptualization; investigation; writing—original draft; writing—review and editing; methodology; formal analysis; resources; data curation. **Francesca Lazzaroni**: Investigation; writing—original draft; writing—review and editing; visualization; methodology; formal analysis; supervision; data curation. **Matteo Claudio Da Via'**: Conceptualization; investigation; funding acquisition; writing—original draft; writing—review and editing; methodology; formal analysis; resources; supervision; data curation. **Marta Lionetti**: Writing—review and editing; methodology; supervision; data curation. **Giulia Maggioni**: Funding acquisition; writing—review and editing; resources. **Alessia Campagna**: Writing—review and editing; resources. **Denise Ventura**: Writing—review and editing; resources. **Giorgio A. Croci**: Writing—review and editing; validation. **Alessio Marella**: Writing—review and editing; formal analysis; software. **Akihiro Maeda**: Writing—review and editing; investigation; formal analysis. **Antonio Matera**: Investigation; writing—review and editing; formal analysis. **Emanuele Calvi**: Writing—review and editing. **Margherita Scopetti**: Writing—review and editing. **Filippo Viviani**: Writing—review and editing. **Nicolò Rampi**: Writing—review and editing; resources. **Davide Bonometti**: Writing—review and editing; resources. **Elena Benzoni**: Writing—review and editing; formal analysis. **Manuela Zappa**: Writing—review and editing; formal analysis. **Sonia Fabris**: Writing—review and editing; formal analysis. **Marzia Barbieri**: Writing—review and editing; formal analysis. **Stefania Pioggia**: Writing—review and editing; formal analysis. **Claudia Leoni**: Writing—review and editing; resources. **Antony Ricchiuti**: Writing—review and editing; resources. **Matteo G. D. Porta**: Funding acquisition; writing—review and editing; resources. **Mariarita Sciumè**: Writing—review and editing; resources. **Francesco Passamonti**: Writing—review and editing; resources. **Nicola S. Fracchiolla**: Writing—review and editing; resources; conceptualization; funding acquisition. **Niccolò Bolli**: Conceptualization; investigation; funding acquisition; writing—original draft; writing—review and editing; resources; supervision; data curation; visualization; project administration.

## CONFLICT OF INTEREST STATEMENT

N.B. received honoraria from Amgen, GSK, Janssen, Jazz, Pfizer, and Takeda. M.C.D.V. served as Advisory Board for Takeda, and on the Speakers Bureau for Janssen, Sanofi, and GSK. F.P. received honoraria during the last two years for lectures from Novartis, Bristol‐Myers Squibb, Abbvie, GSK, Janssen, AOP Orphan, and for advisory boards from Novartis, Bristol‐Myers Squibb/Celgene, GSK, Abbvie, AOP Orphan, Janssen, Karyiopharma, Kyowa Kirin and MEI, Sumitomo, Kartos. Speaking bureau and travel grants from Amgen, Pfizer, JAZZ, Menarini, Serb, Incyte to N.S.F. The remaining authors declare no competing financial interest.

## Supporting information

Supporting information file 1.

## Data Availability

GEO SUBMISSION GSE 315856 Original data for single‐cell RNA sequencing are available on the platform, and the code will be available on request to the corresponding author.
