# Supplementary material for: Immune and cellular determinants of response to HMA‐venetoclax regimens in acute myeloid leukemia
Source: Hemasphere. 2026 Oct 13;10(10):e70498. doi: 10.1002/hem3.70498 (PMC13660835; doi:10.1002/hem3.70498)
Supplement: Supplementary file 1 [file HEM3-10-e70498-s001.docx]

**Immune and Cellular Determinants of Response to HMA-Venetoclax Regimens in Acute Myeloid Leukemia**

**SUPPLEMENTARY MATERIALS AND METHODS**

**Single-Cell 5’ RNA Sequencing**

Thawed MNCs were processed using the Chromium Next GEM Single Cell 5' v2 kit (10x Genomics, PN-1000263). Approximately 9,000 cells per sample were loaded per Chip K (PN-2000286), and libraries were generated for both gene expression and T cell receptor (TCR) enrichment using the Human TCR Amplification Kit (PN-1000252). Full-length cDNA was amplified, purified using SPRIselect (Cat #B23318), and quantified with the Picogreen dsDNA assay (Cat #P7589). Libraries (Dual Index Library Kit, 10X Genomics, PN-1000215) were sequenced on the Illumina NovaSeq 6000 platform (150 bp paired-end reads), targeting 100,000 reads/cell for gene expression and 15,000 reads/cell for TCR.

**Single-Cell RNA Sequencing Pre-processing**

Raw sequencing files were processed with Cell Ranger v5.0 (10x Genomics), aligned to GRCh38, and further cleaned using SoupX to remove ambient RNA. Cells were filtered based on quality metrics: mitochondrial reads <10%, 200–3,000 detected genes, and exclusion of predicted doublets (DoubletFinder v2.0.4, Supplementary Figure 1). Annotated cell types were mapped to the Bone Marrow Reference Map (v4.4.0) and ProjecTILs atlas for T-cell classification.

**Data Integration and Analysis**

Seurat v4.4.0 was used for normalization, identification of variable features, and integration using reciprocal PCA anchoring. Dimensionality reduction was performed with PCA followed by UMAP. Harmony v1.2.0 was applied to correct for batch effects. Differential gene expression was assessed using the Wilcoxon rank-sum test, and cluster-specific markers were identified with FindAllMarkers. Gene set enrichment analysis (GSEA) was conducted using clusterProfiler (adjusted p < 0.1) v4.10.0 and MSigDB Hallmark gene sets via msigdbr v7.5.1. Epithelial to mesenchymal (EMT) module score was calculated using single sample GSEA from the GSVA package v1.50.5. EMT-high cutoff was determined through log-rank statistic maximization and was set at 0.39.

**TCR Repertoire Analysis**

Clonotype and diversity analyses of TCR repertoires were performed using the scRepertoire v2.0 package. Metrics included clonal abundance, diversity indices, and CDR3 length distribution, as previously described [14].

**Data Visualization**

Plots and heatmaps were generated using ggplot2 v3.4.4 and ComplexHeatmap v2.18.0. Summary statistics were plotted in GraphPad Prism v10.0. Figures were composed in Adobe Illustrator 2024, and schematics were designed using BioRender.com.

**SUPPLEMENTARY FIGURES**

**Supplementary Figure 1.** **Quality control metrics across single-cell RNA-seq samples**. **(A)** Violin plots showing the distribution of total RNA counts (nCount_RNA) per cell across all samples. **(B)** Percentage of mitochondrial transcripts (Percent_MT) per cell, used to assess cell viability and stress. **(C)** Number of detected RNA features (nFeature_RNA) per cell, reflecting transcriptome complexity. **(D)** Percentage of hemoglobin transcripts (Percent_HB), indicative of erythroid contamination. Samples are color-coded by condition. These metrics were used to filter low-quality cells prior to downstream analysis.

**Supplementary Figure 2.** **Kaplan-Meier survival analysis stratified by genomic risk and transplant status**. **(A)** Overall survival (OS) curves comparing ELN2024-defined genomic risk groups (Favorable, Intermediate, Adverse) in patients treated with HMA-Venetoclax. Left panel shows significant OS differences in first-line treated patients (*p = 0.001*); right panel shows non-significant differences in later-line treated patients (*p = 0.2***). (B)** Survival outcomes according to IDH1 and NPM1 mutational status. **(C)** OS curves stratified by stem cell transplant (SCT) status and number of prior therapy lines (*p = 0.001*). Tables below each plot indicate number of patients at risk and events over time.

**Supplementary Figure 3.** **Cell recovery across single-cell RNA-seq samples stratified by response and timepoint.** **(A)** Bar plots showing the number of high-quality cells recovered per sample at diagnosis (TP1) and post-cycle one (TP2), stratified by response group: non-responders (NR, orange) and patients achieving composite complete remission (CRi, blue). **(B)** Box plots comparing cell recovery between NR and CRi groups at TP1 and TP2. No statistically significant differences were observed (Wilcoxon test, p > 0.5).

**Supplementary Figure 4. Proportional distribution of annotated immune cell types across response groups and timepoints**. Bar plots show the relative abundance of BM immune populations in non-responders (NR, orange) and patients achieving composite complete remission (CRi, blue) at diagnosis (TP1, left) and post-cycle one (TP2, right). Cell types were annotated using the Bone Marrow Reference Map. Responders exhibit post-treatment expansion of CD8⁺ T cells and early erythroid/megakaryocytic progenitors, while non-responders retain elevated T-cell fractions across timepoints.

**Supplementary Figure 5 A,B.** **Bar plots showing inferred CNV states across diagnostic AML samples (TP1, TP2) using Numbat**. Each panel represents a single patient, with CNV frequencies plotted across selected genomic regions. Color-coded states reflect the myeloid cellular differentiation. Absent TP2 samples reflected no detected CNV abnormalities. Cytogenetic abnormalities obtained from karyotype are displayed in **Supplementary Table 2** at patient level. While AML3 and AML9 had no major cytogenetics abnormalities by karyotype, the Numbat algorithm identified CNVs involving chromosome 4 and chromosome 15 respectively, of unknown significance. Similarly, the Numbat algorithm did not capture i(17)(q10) in AML7, while it identified chromosome 4 abnormalities not identified by karyotype.

**Supplementary Figure 6.** **Dot plot showing expression of selected genes across monocytic, GMP-like, and immature myeloid compartments**. Dot size reflects percentage of expressing cells; color intensity indicates average expression level.

**Supplementary Figure 7. Trajectory inference of myeloid compartments using Slingshot.** **(A)** Pseudotime trajectories at TP1 (black) and TP2 (pink). **(B)** Same trajectories overlaid on annotated myeloid subtypes, including monocytic, GMP-like, and immature populations.

**Supplementary Figure 8. Dot plots showing Hallmark pathway enrichment corresponding to panels A-D of Figure 3**. **(A)** Differential enrichment in non-responders at TP2 versus TP1. **(B)** Baseline enrichment in CRi patients at TP1. **(C)** Comparison of RR versus CRi at TP2. **(D)** Longitudinal changes in CRi patients between TP1 and TP2. Dot size reflects gene count; color indicates adjusted p-value.

**Supplementary Figure 9.** **Immune cell frequencies and IDO1 expression across response groups and timepoints**. **(A)** Bar plots showing absolute frequencies of CD4⁺, CD8⁺, Treg, and proliferating T cell subsets at TP1 and TP2 in non-responders (NR, orange) and CRi patients (blue). **(B)** Violin plots showing IDO1 expression across the same cell types and conditions.

**Supplementary Figure 10.** **Dot plot showing expression of selected TNFα/NF-κB target genes in CRi and NR patients.** Dot size reflects percentage of expressing cells; color intensity indicates average expression level. Genes include MCL1, CCND1, FOS, FOSB, and RHOB, associated with leukemic survival and resistance in non-responders.

**Supplementary Figure 11. *STAT1* expression dynamics across cell subsets and response groups.** *STAT1* expression in dendritic, $\text{CD4}^{+}$ and $\text{CD8}^{+}$ T, monocytic, and immature/intermediate myeloid cells in non-responders (NR) vs. complete remission (CRi) at baseline (TP1) and post-cycle 1 (TP2). Baseline *STAT1* is constitutively higher in NR across most subsets, but this difference attenuates at TP2, most prominently in monocytic cells.

**Supplementary Figure 12.** **Dot plots showing Hallmark pathway enrichment in non-responders (NR) and complete responders (CR) at TP1 and TP2**. **(A)** Comparison of NR and CR at BL. **(B)** Comparison of NR and CR at PD. Enriched pathways include TNFα/NF-κB, IL6/JAK/STAT3, EMT, hypoxia, and KRAS signaling, consistent with inflammatory and stromal remodeling in resistant AML.

**Supplementary Figure 13.** **Kaplan-Meier survival analysis of TCGA AML patients stratified by EMT signature score.** Patients with high EMT scores (magenta) show significantly shorter overall survival compared to EMT-low cases (green). These findings validate the association between EMT activation and poor prognosis, as described in Figure 4D.

**Supplementary Figure 14. Forest plot showing hazard ratios for overall survival in TCGA AML patients stratified by EMT signature and TP53 mutation status.** The reference group is EMT-low/TP53 wild-type. EMT-high/TP53-mutant patients show significantly increased risk (HR p < 0.001 and p = 0.002), while EMT-high/TP53 wild-type cases do not reach significance. These data support the additive prognostic impact of EMT activation within TP53-mutant AML.

**Supplementary Figure 15.** **Violin plots showing expression of anti-apoptotic genes BCL2 (A), BCL2L1 (B), and BCL2A1 (C) across myeloid compartments in CRi (blue) and NR (orange) patients at diagnosis (TP1) and post-treatment (TP2).** Expression is stratified by cell type: Monocyte, Macrophage, Intermediate, Immature, and Other. Non-responders show elevated BCL2A1 post-treatment, while responders exhibit increased BCL2L1, consistent with divergent apoptotic remodeling described in Figure 4F.

**Supplementary Figure 16.** **Violin plots showing expression of immune checkpoint genes LAG3 (A), PDCD1 (B), HAVCR2 (C), and TIGIT (D) across CD8⁺ T cell subsets in CRi and NR patients.** Cell types include Naive, Central Memory (CM), Effector Memory (EM), TEMRA.

**SUPPLEMENTARY TABLES**

**Supplementary Table 1.** Clinical characteristics, outcomes and ELN2024 classification of 132 patients treated with HMA+Venetoclax in either first or later lines of therapy. *ELN2024 classification could be established in a subset of patients. This score is currently validated only in patients treated in the first line setting.

|  | **First line treatment** | **>1 lines of treatment** |
| --- | --- | --- |
| **n** | 65 | 67 |
| **Age (IQR)** | 75.3 (71.8-78.22) | 64.9 (56.0-72.1) |
| **CRi** | 51 (78.4%) | 34 (50.8%) |
| **HSCT*** | 9 (13.9%) | 43 (26.9%) |
| **mOS (months)** | 8.68 | 6.09 |
| **ELN 2024**** | Available in 51/65 | Available in 49/67 |
| **Favorable** | 32 (62%) | 25 (51%) |
| **Intermediate** | 12 (23.5%) | 13 (26%) |
| **Adverse** | 7 (13.7%) | 11 (22.4%) |

**Supplementary Table 2**. Presenting features of n=10 patients with de novo AML according to treatment allocation.

|  | **Decitabine+Venetoclax** | **Azacitidine+Venetoclax** |
| --- | --- | --- |
| **n** | 7 | 3 |
| **Median age** | 70 | 75 |
| **Sex (M %)** | 57 | 66 |
| **White blood cell count WBC (mm^3^)** | 4880 (1490-68900) | 3455 (740-14360) |
| **Hemoglobin (g/dl)** | 9 (8.7-13) | 9.2 (8.4-10.1) |
| **Platelets (mm^3)^** | 51.000 (34.000-135.000) | 97.000 (25.000-144.000) |
| **BM blasts (%)** | 22.5 (18-80) | 53 (25-80) |
| **Response status (at any timepoint)** | | |
| **Complete response w/o count recovery (%)** | 57.2 (4) | 67 (2) |
| **Non Response (%)** | 14.3 (1) | 33 (1) |
| **Refractory disease (%)** | 28.5 (2) |  |
| **Genomic defining lesion** | | |
| **NPM1** |  | 1 |
| **Chromatine-spliceosome complex mutation** | 5 | 2 |
| **TP53/CK** | 1 |  |
| **Other** | 1 |  |
| **Karyotype** | | |
| **AML1** | 47,XY,t(6;17)(q31;p13)?c,+8[20].ish 17p13(TP53x2)[10] |  |
| **AML3** |  | 46 XY [20] |
| **AML5** | 46,XX,i(17)(q10)[11]/46,XX[9] |  |
| **AML7** |  | 46,XY,del(20)(q11.2q13.2)[4]/46,XY[18] |
| **AML9** | 46, XX[20] |  |
| **AML11** |  | 46,XX,del(5)(q13q32-34)[20] |
| **AML14** | 47,XY,+8[20] |  |
| **AML16** | 47,XY,+8[19]/46,XY[4] |  |
| **AML18** | 46,XY[20] |  |
| **AML20** | 43-45,XX,add(1)(q43-44),der(3)t(3;?)(q12;?),del(5)(q13q33),-15,add(15)(p11.1),der(16)t(16;?)(q21;?),-17, add(18)(q23),del(20)(q11.2-13.1q11.2-13.1),-22,-22,+r,+mar1,+mar2[cp6]/46,XX[14] |  |

**Supplementary Table 3.** Absolute count of CD4 T cells with unique TCR observed at both timepoints

| **Patient** | **Timepoint 1 (TP1)** | **Timepoint 2 (TP2)** |
| --- | --- | --- |
| AML1 (RR) | 0 | 1 |
| AML3 (NR) | 3 | 4 |
| AML5 (CRi) | 1 | 0 |
| AML7 (CRi) | 89 | 145 |
| AML9 (CRi) | 4 | 156 |
| AML11 (CRi) | 35 | 55 |
| AML14 (CRi) | 0 | 0 |
| AML16 (CRi) | 11 | 13 |
| AML18 (NR) | 13 | 14 |
| AML20 (RR) | 49 | 41 |

**Supplementary Table 4**. Absolute count of CD8 T cells with unique TCR observed at both timepoints

| **Patient** | **Timepoint 1 (TP1)** | **Timepoint 2 (TP2)** |
| --- | --- | --- |
| AML1 (RR) | 41 | 62 |
| AML3 (NR) | 155 | 251 |
| AML5 (CRi) | 2 | 1 |
| AML7 (CRi) | 1174 | 1465 |
| AML9 (CRi) | 25 | 181 |
| AML11 (CRi) | 362 | 620 |
| AML14 (CRi) | 65 | 16 |
| AML16 (CRi) | 182 | 1115 |
| AML18 (NR) | 236 | 343 |
| AML20 (RR) | 1590 | 1362 |
